# Widespread prevalence of plasmid-mediated *bla*_CTX-M_ type extended-spectrum beta-lactamase *Escherichia coli* in backyard broiler production systems in the United States

**DOI:** 10.1371/journal.pone.0304599

**Published:** 2024-06-03

**Authors:** Jessica L. Parzygnat, Rocio Crespo, Matthew D. Koci, Robert R. Dunn, Lyndy Harden, Mary Fosnaught, Siddhartha Thakur

**Affiliations:** 1 Department of Population Health and Pathobiology, North Carolina State University College of Veterinary Medicine, Raleigh, NC, United States of America; 2 Prestage Department of Poultry Science, North Carolina State University, Raleigh, NC, United States of America; 3 Department of Applied Ecology, North Carolina State University, Raleigh, NC, United States of America; Universidad San Francisco de Quito, ECUADOR

## Abstract

Extended-spectrum beta-lactamase (ESBL) *Escherichia coli* (*E*. *coli*) is an emerging pathogen of high concern given its resistance to extended-spectrum cephalosporins. Broiler chicken, which is the number one consumed meat in the United States and worldwide, can be a reservoir of ESBL *E*. *coli*. Backyard poultry ownership is on the rise in the United States, yet there is little research investigating prevalence of ESBL *E*. *coli* in this setting. This study aims to identify the prevalence and antimicrobial resistance profiles (phenotypically and genotypically) of ESBL *E*. *coli* in some backyard and commercial broiler farms in the U.S. For this study ten backyard and ten commercial farms were visited at three time-points across flock production. Fecal (n = 10), litter/compost (n = 5), soil (n = 5), and swabs of feeders and waterers (n = 6) were collected at each visit and processed for *E*. *coli*. Assessment of ESBL phenotype was determined through using disk diffusion with 3^rd^ generation cephalosporins, cefotaxime and ceftazidime, and that with clavulanic acid. Broth microdilution and whole genome sequencing were used to investigate both phenotypic and genotypic resistance profiles, respectively. ESBL *E*. *coli* was more prevalent in backyard farms with 12.95% of samples testing positive whereas 0.77% of commercial farm samples were positive. All isolates contained a *bla*_CTX-M_ gene, the dominant variant being *bla*_CTX-M-1_, and its presence was entirely due to plasmids. Our study confirms concerns of growing resistance to fourth generation cephalosporin, cefepime, as roughly half (51.4%) of all isolates were found to be susceptible dose-dependent and few were resistant. Resistance to non-beta lactams, gentamicin and ciprofloxacin, was also detected in our samples. Our study identifies prevalence of *bla*_CTX-M_ type ESBL *E*. *coli* in U.S. backyard broiler farms, emphasizing the need for interventions for food and production safety.

## Introduction

Extended-spectrum beta-lactamases (ESBL) are enzymes that confer resistance to a commonly used group of antibiotics in medicine, beta-lactams, such as penicillin and cephalosporins. Pathogens that produce ESBL are known for their resistance to cephalosporins, particularly extended-spectrum (3rd generation) cephalosporins that were developed to cover a wider range of Gram-negative pathogens [1, 2]. Public health concerns regarding resistance to these vital antimicrobial therapies have greatly increased because of ESBL spread worldwide. Extended-spectrum beta-lactamase (ESBL) producing Enterobacterales, such as *Escherichia coli*, are listed as a “serious threat” to public health in the CDC’s 2019 Antimicrobial Resistance Threats report for the United States [3]. While the increased prevalence of ESBL pathogens in hospital settings is of concern, prevalence has also been associated with food animals, particularly broilers [1, 4–6]. Broilers are chickens grown for meat consumption and are known for high rates of ESBL *E*. *coli* contamination [6–8]. This is alarming as broiler chicken is the number one consumed meat in the United States and worldwide [9, 10]. Given the large “flux” of chickens into the market and hence domestic kitchens, even low prevalence microbes have the potential to have large and ramifying effects.

The production of broiler chickens is dominated by integrated commercial production; however, backyard poultry ownership has increased in the United States [11–13]. The USDA defines backyard poultry farms as those that have less than 1,000 birds [14]. In some cases, these birds are kept as pets and are not consumed. In others, they are consumed exclusively by the owners. In others, meat products or eggs are still produced by the birds and may be sold to customers from the backyard farm or at a local farmers market. This growth in ownership of backyard chickens has outpaced our understanding of the identity, prevalence, and ecology of antimicrobial-resistant (AMR) pathogen prevalence in U.S. backyard farm environments [11]. For example, our understanding of ESBL *E*. *coli* prevalence and resistance in backyard broiler production in the United States is minimal, which is concerning considering potential transfer of ESBL *E*. *coli* from broilers to farm owners, or others in the home [15]. The primary public contact with commercially produced chickens is as butchered and packaged meat; however, owners of backyard chickens have the potential for more points of contact. Elkhoraibi and colleagues (2014), conducted a survey and found many United States poultry owners considered their birds to be “safer” to consume [11]. This mindset could cause decreased standards for biosecurity and hygiene protocols, leading to increased risk of pathogen exposure [11]. Though there are few backyard studies in the United States, at least some backyard chickens can host extensively resistant organisms such as ESBL *E*. *coli*. For example, a Shah and colleagues found ESBL *E*. *coli* in 29% of the backyard poultry farms they tested in Washington [16]. Without a better understanding of ESBL *E*. *coli* prevalence in these U.S. farm systems, it is difficult to develop anticipatory interventions, guidance, guidelines or policies.

We sought to compare the AMR profiles of commercial and backyard chickens in a portion of the Southeastern United States. Our longitudinal study follows birds in backyard and integrated commercial farm flocks and details the prevalence and AMR profiles (phenotypic and genotypic) of ESBL *E*. *coli*. Our study aims to better understand the prevalence of ESBL *E*. *coli* in these production systems in order to lay groundwork for improving food and production safety.

## Methods

### Farm

Backyard farms in our study are considered to be farms that raise broilers at a residential property for meat consumption, either by the household or sold to the public for consumption at local farmers markets or the farm itself. Criteria for inclusion were that birds are broiler breeds raised for meat consumption. In this study, all backyard flocks were Cornish Cross. Few similarities were noted, but all farms did not have birds from the same hatchery or acquire the same feed. Farms varied in litter management (though the rule for almost all farms was new flock starts on new litter), cleaning protocols, and feed/water supplementation. Flocks varied from 22 to 1,000 birds in a flock. Commercial integrated flocks are broilers raised for meat consumption as part of large commercial companies. All commercial integrated flocks were raised in indoor intensive flocks. No other species were housed on these farms. Flocks contained 13,500 to 30,900 birds in a flock (mean flock size = 20,630 birds). Farm management such as litter management and feed/water supplementation mostly depending on commercial company of origin. All commercial flocks contained Ross 708 broilers. These farms will be referred to as commercial for the rest of the paper.

Ten backyard flocks from 8 farm locations and ten commercial flocks from ten different houses from ten different locations. For the commercial farms, the flocks sampled were split amongst three different companies (Company One = 2 flocks, Company Two = 2 flocks, Company Three = 6 flocks). Backyard farms were all different, except farm 1 and 7 and farm 5 and 9. These two pairs were the same farm, but sampled in a different season (ex: first visit spring and second visit fall). Flocks were sampled at brooding, grow out, and finishing in order to encompass all production stages. These three timepoints throughout production fell at days 10, 31, and 52 for backyard farms and days 10, 24, and 38 for commercial farms. Commercial integrator farms were sampled at a shorter timeline because birds were processed faster than they are in the backyard environment. All sampling occurred between April 2021 and April 2023.

At each farm visit, fecal (n = 10), soil (n = 5), litter or compost (n = 5), and feeder and waterer swabs (n = 6), were collected at each visit. The litter or compost sample depended on what was available at the farm visit. All commercial samples under this category were litter, given it was always available. For backyard farms typically birds were on litter for the first visit, but were on pasture for the remaining. Therefore, compost samples were collected instead, as many owners put old litter samples into their compost piles.

The North Carolina State University Institutional Animal Care and Use Committee (IACUC) reviewed and approved the work done with broilers for this study. The IACUC approval number is: 20–249.

### Isolation of ESBL *E*. *coli*

The protocol for isolating ESBL *E*. *coli* was a modified version of the 2021 National Antimicrobial Resistance Monitoring System protocol [17]. To isolate *E*. *coli*, 90 mL of buffered peptone water was poured on to each sample in sterilize closure bags. The sterilize closure bag was then placed in an automatic shaker at 200 rpm for 15 minutes, and then incubated overnight at 35°C. After incubation, the bag was massaged before using a 10 μL inoculation loop to t-streak the buffered peptone water and sample mixture onto a MacConkey plate with 4 μg/mL of Cefotaxime. One plate was created per sample. Plates were placed in an incubator at 35°C for 24 hours. After incubation, a 1 μL loop was used to grab a pink colony, briefly swirl it in a 5mL tube of tryptone water, and then immediately plate on blood agar. One colony was collected per plate. The inoculated tryptone tubes and blood agar were incubated at 35°C for 24 hours. The tryptone tubes were used to conduct an indole test by placing 5 drops of Kovac’s reagent on the inner side of the tube so it slides into solution. Tubes that created a pink ring with the addition of Kovac’s reagent were deemed *E*. *coli* and were tested for the ESBL phenotype.

### ESBL testing

Processed isolates were assessed through a disc diffusion for the ESBL phenotype through the procedures explained in Jacob et al., 2020 [18]. First, a 0.5 McFarland suspension was created with a 24 hour fresh culture on blood agar. A cotton tip swab was then used to create a lawn of bacteria across a Mueller Hinton agar plate. The four antibiotic disks (Cefotaxime 30 μg, Cefotaxime with Clavulanic acid 30 μg, Ceftaxidime 30 μg, Ceftazidime with Clavulanic acid 30 μg) were separated into quadrants of the plate. After incubation at 35°C, the zone of inhibition surrounding the antibiotic disks was measured. If the zone of inhibition surrounding the antibiotic disc with either cefotaxime or ceftazidime (3^rd^ generation cephalosporins) alone was at least 5 mm larger than the zone of inhibitions surrounding the disk with the 3^rd^ generation cephalosporin with clavulanic acid (beta lactamase inhibitor), the isolate was deemed to be ESBL.

### Antimicrobial susceptibility testing

Each isolate deemed to have the ESBL phenotype was tested using the Thermo Scientific Sensititre ™ ESB1F plates. Testing was conducted by following the National Antimicrobial Resistance Monitoring System (NARMS) 2020 Manual of Laboratory Methods for *E*. *coli* [19]. Briefly, the isolate was used to create a 0.5 McFarland suspension using 5 mL tube of demineralized water. Next, 10 μL of the suspension was placed in 11 mL of Mueller Hinton Broth and vortexed. The sensititre machine (Thermo Fisher) was then used to place 50 μL of the Mueller Hinton with isolate suspension into the ESBF sensititre plate. The antimicrobials tested by the plate are: ceftazidime (TAZ; 0.25–128 μg/mL), cefazolin (8–16 μg/mL), cefepime (FEP; 1–16 μg/mL), cefoxitin (FOX; 4–64 μg/mL), cephalothin (CEP; 8–16 μg/mL), cefpodoxime (POD; 0.25–32 μg/mL), cefotaxime (FOT; 0.25–64 μg/mL), ceftriaxone (AXO; 1–128 μg/mL), imipenem (IMI; 0.5–16 μg/mL), meropenem (MERO; 1–8 μg/mL), gentamicin (GEN; 4–16 μg/mL), ampicillin (AMP; 8–16 μg/mL), ciprofloxacin (CIP; 1–2 μg/mL), piperacillin/tazobactam constant 4 (P/T4; 4/4-64/4 μg/mL), ceftazidime/clavulanic acid (T/C; 0.12/4-128/4 μg/mL), and cefotaxime/clavulanic acid (F/C; 0.12/4-64/4 μg/mL). Plates were covered and placed in the incubator at 35.0°C for 24 hours. After incubation, the plates were viewed using a Manual Viewbox (Thermo Fisher). Minimum inhibitory concentration (MIC) was determined by identifying the antimicrobial concentration that inhibited bacterial growth. The ESBL phenotype was also confirmed through broth microdilution according to CLSI guidelines. Breakpoints for susceptible, intermediate, resistant, and susceptible dose-dependent were determined through combined CLSI and NARMS breakpoints [20, 21]. A three two-fold dilution for either cefotaxime with clavulanic acid or ceftazidime with clavulanic acid was needed to count as ESBL, in accordance with CLSI guidelines [20].

### DNA isolation and whole genome sequencing

Bacterial genomic DNA was extracted using the Qiagen DNeasy PowerLyzer Microbial Kit (Qiagen, Hilden, Germany) according to the manufacturer’s recommendation. A NanoDrop 2000/2000c Spectrophotometer (Thermo Fisher Scientific, Waltham, Massachusetts) and a Qubit Flex Fluorometer (Thermo Fisher Scientific) were used to deter mine the concentration and quality of DNA. DNA libraries were prepared using the Illumina DNA Prep Kit (Illumina, San Diego, California) according to manufacturer’s instructions. The DNA libraries were then re-quantified using the Qubit Flex Fluorometer. Sequencing was performed on the Illumina MiSeq System using MiSeq Reagent Kit v3 600 cycles (Illumina). All isolates can be found under the BioProject number: PRJNA293225.

### Bioinformatic pipeline

This project used the same pipeline that is described in Hull et al, 2021 [22]. Whole genome sequence forward and reverse reads (fastq) were assembled (fasta) *de novo* with Shovill v1.1.0 using SPAdes v3.15.2 [23]. The quality of genome assembly was assessed with QUAST v5.0.2 and the resulting fasta files were blasted against AMR databases through ABRICATE version 1.0.1 [24, 25]. *De novo* assembly of forward and reverse reads was repeated for plasmid detection using Shovill and PlasmidSPAdes genome assembler v3.15.2. Plasmid fasta files were also further analyzed for antimicrobial resistance genes, virulence factors, and plasmid replicons using ABRICATE v1.0.1 [25]. ABRICATE databases run for whole genome fasta files and plasmid fasta files include CARD, MEGAres, VFDB, and Plasmidfiner [26–30]. Amazon Web Service was utilize for computation and storage of data.

### Phylogenetic tree

The phylogenetic tree was assembled through using online open-access tool REALPHY v1.13 [31]. Tree information and other relevant metadata were downloaded into Interactive Tree of Life (iTOL) for visualization [32].

### Statistics

CDC EpiCalc Info StatCalc v5.5.11 software was used to conducting Fisher’s exact two-tailed test when appropriate [33]. Prevalence was determined through counting the total number of positive samples and dividing by the total number of samples collected within a farm type (backyard/commercial).

## Results

**Fig 1:**
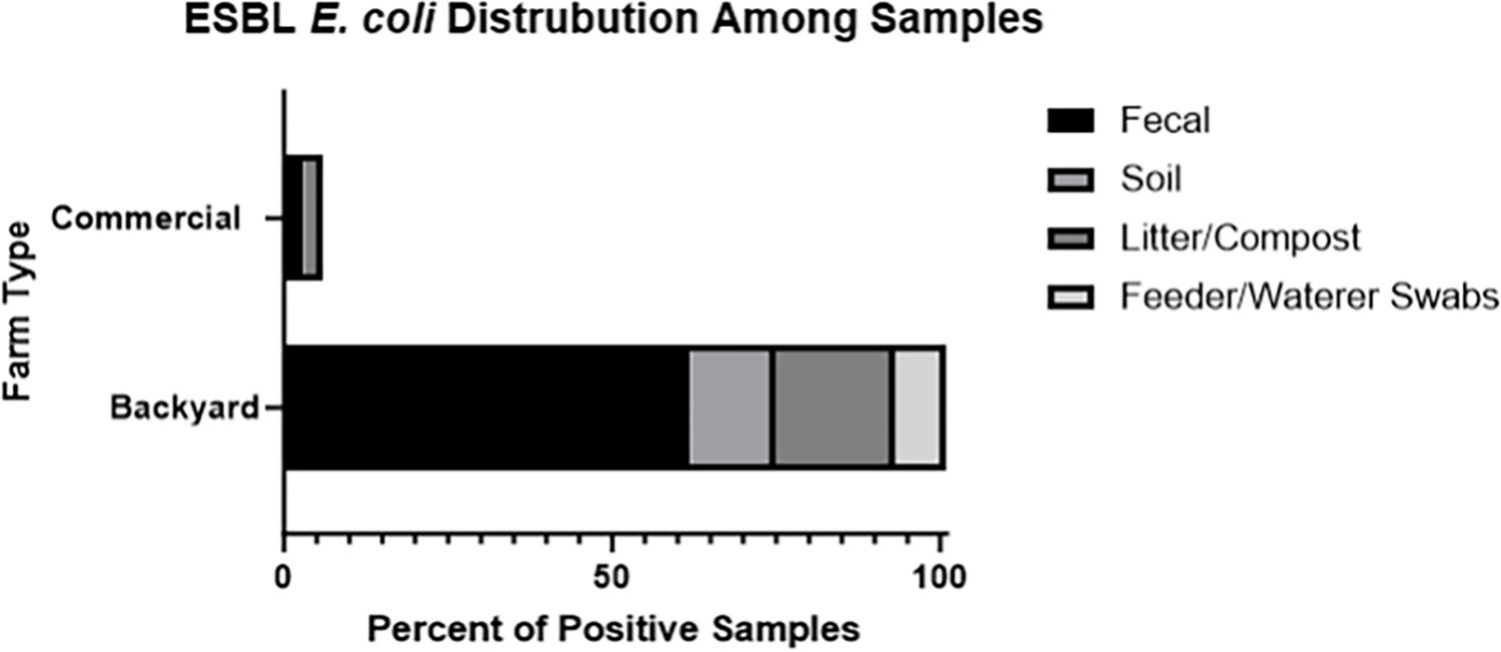
ESBL E. coli sample distribution. Displays the sources of where positive samples were found. The number of isolates corresponding to each sample type are displayed by bar graph height.

Overall, we found 12.95% (n = 101/780) of our backyard farm samples and 0.77% (n = 6/780) of commercial farm samples tested positive for ESBL *E*. *coli*. This fifteen fold difference in prevalence between backyard and commercial farms was statistically significant (Fisher’s Exact Test: p-value < 0.05) with ESBL *E*. *coli* more common in backyard farms. Prevalence was found in eight of the ten backyard flocks sampled but in just two of the ten commercial farms. In terms of sample type, a little over half came from fecal samples (61.4%; n = 62/101) and the rest came from environmental sources (soil: 12.87%, n = 13/101; litter/compost: 17.82%, n = 18/101; feeder and waterer swabs: 7.92%, n = 8/101) (Fig 1). Commercial farms positive samples were found in fecal (50%, n = 3/6) and litter (50%, n = 3/6) samples. All flocks, backyard and commercial, that tested positive in litter samples also tested positive in fecal samples on the same visit. All soil samples that tested positive also had fecal samples test positive in the same visit, except for one sample. This soil sample tested positive in the second visit, but the there was only one positive fecal sample in the first visit. In addition, two waterer swabs tested positive in two different farms that did not have any other samples test positive in any other visit.

The individual isolates of ESBL *E*. *coli* differed with regard to the antimicrobials which they were resistant and the “generation” of those antimicrobials. Of importance with ESBL pathogens are generations of cephalosporins. Cephalosporins come in 5 generations, each later generation improving the spectrum of infections which it can cover, particularly when it comes to improving action against Gram-negative pathogens [34]. Antimicrobial susceptibility testing revealed expected phenotypic resistance to 3rd generation cephalosporins, but concerning resistance to later 4^th^ generation cephalosporin, cefepime. All isolates from both backyard and commercial farms were resistant to 1^st^ generation cephalosporins, cefazolin and cephalothin. Resistant and intermediate isolates were found to 2^nd^ generation cephalosporin, cefoxitin, in backyard farms (resistant n = 3/101; intermediate n = 15/101). One intermediate isolate to cefoxitin was found in commercial farms. All isolates from backyard and commercial farms were resistant to 3^rd^ generation cephalosporins, cefotaxime, cefpodoxime, and ceftriaxone. In backyard farms 16.83% (n = 17/101) of isolates were resistant and 1.98% (n = 2/101) isolates were intermediate, or have an unknown therapeutic effect, to 3^rd^ generation cephalosporin ceftazidime [35]. Farms. All commercial isolates (n = 6/6; 100%) were susceptible dose-dependent to 4^th^ generation cephalosporin, cefepime. Susceptible dose-dependent isolates are said to be clinically effective only if a higher drug exposure is used [20]. In backyard farms, there was a mix of susceptible (n = 35/101; 34.65%), susceptible dose-dependent (n = 49/101; 48.51%), and resistant (n = 17/101; 16.83%) isolates to cefepime. All commercial isolates and almost all backyard isolates (n = 99/101; 98.02%) were resistant to ampicillin (penicillin), and the rest were intermediate (n = 2/101; 1.98%). All isolates in backyard and commercial farms were susceptible to carbapenems, imipenem and meropenem, as well as a penicillin with beta-lactamase inhibitor, penicillin-tazobactam. In backyard isolates, few were resistant to non-beta lactams, gentamicin (aminoglycoside, n = 3/101) and ciprofloxacin (fluoroquinolone, n = 3/101). All MIC results are listed in Table 1 (Table 1). Overall, antimicrobial susceptibility testing revealed resistance mostly characteristic of ESBL pathogens, except for resistance to 4^th^ generation cephalosporin, cefepime.

**Table 1:** Commercial and backyard ESBL *E*. *coli* squashtogram.

			Distribution of MIC (ug/mL) (# of isolates)											
Antimicrobial^a^	Source	% resistant	0.12	0.25	0.5	1	2	4	8	16	32	64	128	256
AMP	Commercial	98.02%							0	2	**99**			
	Backyard	100%							0	0	**6**			
P/T4	Commercial	-						101	0	0	0	0		
	Backyard	-						6	0	0	0	0		
FAZ	Commercial	100%							0	0	**101**			
	Backyard	100%							0	0	**6**			
CEP	Commercial	100%							0	0	**101**			
	Backyard	100%							0	0	**6**			
FOX	Commercial	2.97%						45	38	15	0	0	**3**	
	Backyard	-						4	1	1	0	0		
FOT	Commercial	100%		0	0	0	0	0	**11**	**42**	**20**	**12**	**16**	
	Backyard	100%		0	0	0	0	0	0	**1**	**3**	**2**		
F/C	Commercial	-	98	0	0	0	1	2	0	0	0	0		
	Backyard	-	6	0	0	0	0	0	0	0	0	0		
TAZ	Commercial	16.83%		4	17	33	19	9	2	**17**	0	0	0	
	Backyard	-		0	0	5	1	0	0	0	0	0	0	
T/C	Commercial	-	65	34	2	0	0	0	0	0	0	0	0	
	Backyard	-	5	1	0	0	0	0	0	0	0	0	0	
POD	Commercial	100%		0	0	0	0	0	**2**	**2**	**17**	**80**		
	Backyard	100%		0	0	0	0	0	0	0	**1**	**5**		
AXO	Commercial	100%				0	0	0	**2**	**17**	**37**	**23**	**6**	**16**
	Backyard	100%				0	0	0	0	**1**	**1**	**3**	**1**	
FEP	Commercial	16.83%				22	13	43	6	**3**	**14**			
	Backyard	-				0	0	6	0	0				
CIP	Commercial	2.97%				98	**3**							
	Backyard	-				6	0							
GEN	Commercial	2.97%						98	0	**3**				
	Backyard	-						6	0	0				
IMI	Commercial	-			101	0	0	0	0	0				
	Backyard	-			6	0	0	0	0	0				
MERO	Commercial	-				101	0	0	0					
	Backyard	-				6	0	0	0					

The plate range of antibiotic concentration is displayed by the white regions. The numbers indicate the number of isolates displaying the MIC. Bold numbers indicate isolates considered to be resistant. If a number is displayed in the gray region, it means the MIC is greater than the plate concentration range.

^a^Antimicrobials: AMP, Ampicillin; P/T4, Piperacillin/tazobactam; FAZ, Cefazolin; CEP, Cephalothin; FOX, Cefoxitin; FOT, Cefotaxime; F/C, Cefotaxime/clavulanic acid; TAZ, Ceftazidime; T/C, Ceftazidime/clavulanic acid; POD, Cefpodoxime; AXO, Ceftriaxone; FEP, Cefepime; CIP, Ciprofloxacin; GEN, Gentamicin; IMI, Imipenem; MERO, Meropenem.

Our research detected genes associated with extended-spectrum beta-lactamase enzymes, which can confer resistance to beta-lactam antimicrobials in the previous paragraph. All isolates contained one of the following extended-spectrum beta-lactamase genes: *bla*_CTX-M-1_ (n = 61/107; 57.00%), *bla*_CTX-M-15_ (n = 2/107; 1.87%), *bla*_CTX-M-55_ (n = 21/107; 19.63%), and *bla*_CTX-M-65_ (n = 23/107; 21.50%). All six commercial isolates had the *bla*_CTX-M-1_ detected. Other beta-lactamase genes, not extended-spectrum, were also detected and: *ampC* (n = 107/107; 100%), *ampC1* (n = 89/107; 83.17%), and *TEM-1* (n = 22/107; 20.56%). Beta-lactamase *ampC* is associated with cephalosporin, penicillin, and even beta-lactamase inhibitor resistance [36]. *TEM-1* is known to be responsible for 90% of ampicillin resistance in *E*. *coli* [26].

Other genes associated with specific non-beta lactam antimicrobials were also identified. Aminoglycoside resistance genes *aac(3)-IIe* (n = 3/107; 2.80%), *ant(3’’)-IIa* (n = 3/107; 2.80%), *aph(3’’)-Ib* (n = 9/107; 8.41%), *aph(3’)-Ia* (n = 19/107; 17.76%), and *aph(6)-Id* (n = 12/107; 11.21%) were detected. Three isolates that contained the same three aminoglycoside resistance genes (*aac(3) -IIe*, *ant(3’’)-IIa*, and *aph(3’)-Ia)* were the only isolates that displayed phenotypic resistance to gentamicin (n = 3/107; 2.80%). All three of these isolates were from the same farm visit. Ciprofloxacin resistance was noted in three isolates; however, it is uncertain whether more isolates were also resistant. The plate layout concentrations do not allow for accurate determination of resistance or susceptibility at lower concentrations given NARMS/CLSI breakpoints. However, we were able to definitively identify the three resistant isolates and all contained various point mutations that confer resistance to ciprofloxacin. Two of these isolates contained *gyrA_D87N* and *gyrA_S83L* point mutations which are known to confer fluoroquinolone resistance [37].

**Fig 2:**
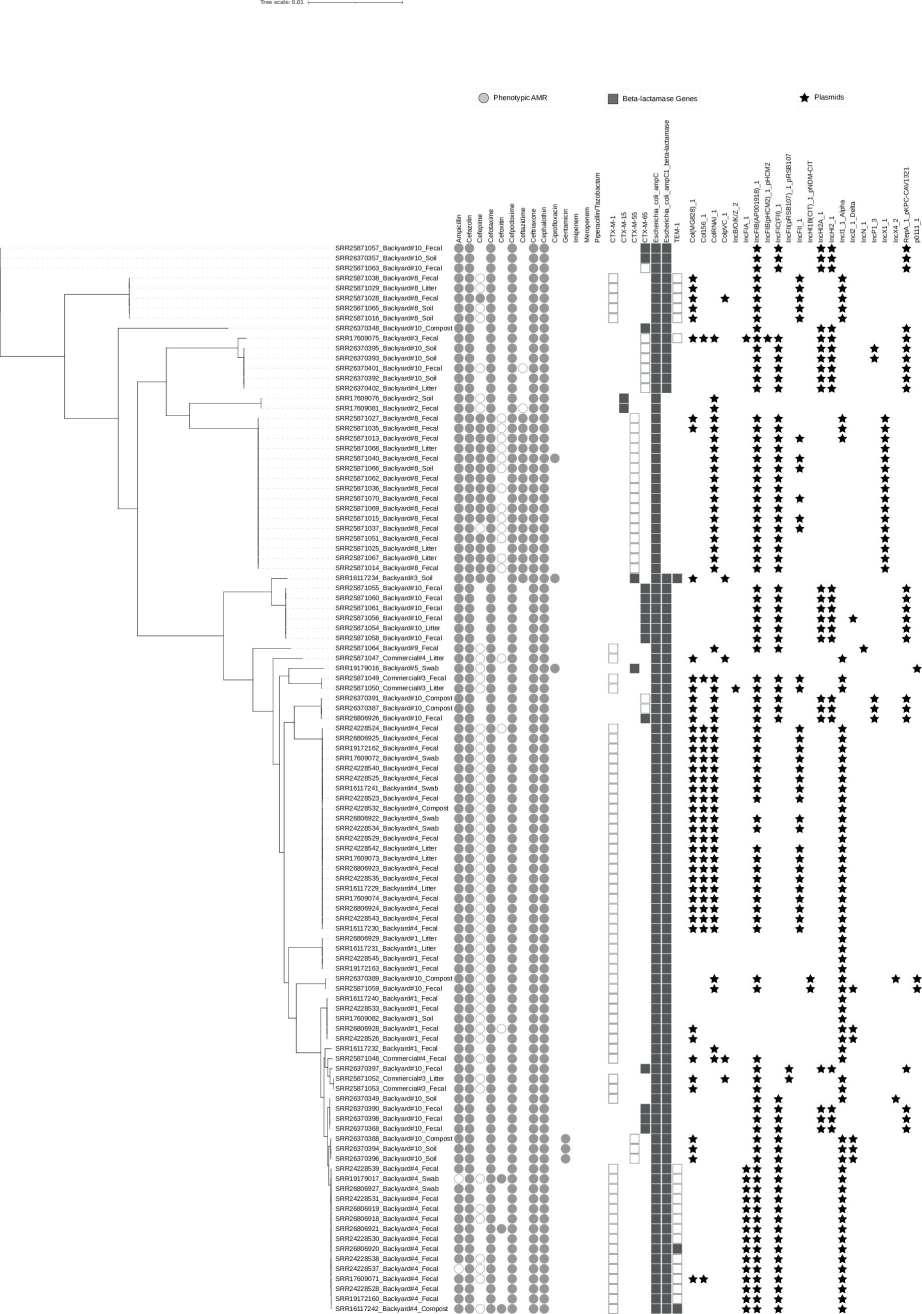
Phenotypic and genotypic characterization dendrogram. Details the phenotypic resistance and genotypic resistance as well as phylogeny of the collected ESBL *E*. *coli* isolates. The isolate labeling on the dendrogram is as follows: SRR#_Farm Type and number_Sample Source. Phenotypic resistance to the antimicrobials listed is represented with a solid circle, intermediate is represented by an open circle, and no circle indicates susceptibility. For cefepime, an open circle indicates susceptible dose-dependent, as opposed to intermediate. Whole genome AMR gene presence is indicated by a square. Open squares indicate the gene was detected in plasmid data. Plasmid detection is indicated by a star.

All isolates contained at least one plasmid and 88.79% (n = 95/107) isolates contained plasmids with detectable antimicrobial resistance genes. The top three plasmids detected are: IncFIB(AP001918)_1 present in 84.11% (n = 90/107), IncI1_1_Alpha in 61.68% (n = 66/107), and IncFIC(FII)_1 in 55.14% (n = 59/107). Of the beta-lactamase associated genes, *bla*_CTX-M-1_, *bla*_CTX-M-55_, *bla*_CTX-M-65_, and *TEM-1* were present in plasmids. All *bla*_CTX-M-1_ genes are present due to plasmids as well as 90.48% (n = 19/21) of *bla*_CTX-M-55_ genes, 39.13% (n = 9/23) of *bla*_CTX-M-65_ genes, and 85.71% (n = 18/21) of *TEM-1* genes. After plasmid assembly a single *CMY-59* gene, correlated with a beta-lactamase, was identified that was not detected in the whole genome sequencing data [26]. This discrepancy is probably due to gene identification after plasmid assembly, but is important to note as it is a beta-lactamase gene [38]. The three gentamicin resistant isolates containing aminoglycoside resistance genes *aac(3) -IIe*, *ant(3’’)-IIa*, and *aph(3’)-Ia*, were all found on plasmids. All phenotypic, genotypic, and plasmid data described above are displayed in Fig 2.

## Discussion

Extended-spectrum beta-lactamase (ESBL) producing bacteria, including *E*. *coli*, are emerging pathogens of high concern given their resistance to beta-lactams, the most commonly used group of antibiotics in medicine [39]. Studies have also noted the concern of zoonotic spread of ESBL *E*. *coli* from poultry to humans and vice versa, which heightens the need to better understand this bacteria given the increase in backyard poultry ownership [16, 40–42]. Studies conducted in outside of the United States (India, Nepal, and Brazil) and indicate varying results when it comes to commercial or backyard systems containing a higher load of ESBL pathogens [40, 43, 44]. In our case, a higher prevalence was reported in backyard farms. Backyard farm studies conducted in other countries attribute misuse of antibiotics as a potential reason for prevalence in this setting [8, 45]. However, as evidenced by our current study and the Shah et al. study [4], antibiotic use is not a requirement for ESBL *E*. *coli* prevalence in poultry farm systems. In fact, our study detected resistance to later generation cephalosporins (4^th^ generation, cefepime) as well as antimicrobials used in treatment of certain ESBL infections, despite no use of antibiotics on the backyard chickens we studied. We identified ESBL *E*. *coli* in both bird fecal samples and farm environment, highlighting the need to better understand this pathogens epidemiology in order to promote production and food safety.

A key question in thinking about the ESBL pathogens in chicken ecosystems is that of the origin of these microbes. This can be considered in proximate or more ultimate contexts. In a proximate context, the first question is where these pathogens were found. Our findings suggest a potential interplay between the chickens and their immediate environments with regard to ESBL *E*.*coli*. A larger proportion of positive samples came from fecal samples as opposed to environmental samples, indicating broilers themselves can be a reservoir of ESBL *E*. *coli* which could therefore spread into the farm environment. All farm visits where litter samples tested positive, fecal samples did as well. Given almost all backyard farms place fresh litter with each new flock of birds, this supports farm environmental contamination could be due to the birds. It has been estimated that hatcheries can be a potential source of beta-lactam resistance genes in *E*. *coli* [46]. In this study, two pairs of flocks came from the same hatchery. In each case, one flock had fecal contamination with ESBL E. coli whereas the other did not, though they came from the same hatchery. Although these birds were purchased from the same hatchery, the purchases occurred at different time periods (18 months difference at least). Transportation of birds to the farm could also be a source of contamination [47]. We recognize this is a small sample size and our study does not resolve the more ultimate questions of origin of these microbes, but indicates the need for further studies investigate different batches of birds from the same hatchery or how transportation could affect pathogen prevalence.

Though the birds themselves could be a source of ESBL *E*. *coli*, our data suggest this may not always be the case. In backyard farms there were two water samples that tested positive when no other samples tested positive from any visit on those two farms. In addition, a soil sample tested positive in the second visit to a farm, in which no fecal samples tested positive in that same visit. We hypothesize bird exposure to other species could be playing and integral role. A unique difference between backyard and commercial farms is that all backyard farms we sampled had other species such as dogs, cats, caprine, bovine, swine, etc. These other species on the farm may not have antimicrobial restrictions. Dogs, cats, bovine, and swine can also be a source of ESBL *E*. *coli* and sharing the same farm environment could cause broiler exposure [48–51]. In many of the backyard farms, other animal species could have close contact with the broilers which raises concern of potential zoonotic transmission. From there, infected broilers could be further contaminating the environment. In addition, exposure to wildlife or different water types have been noted as potential sources for spread of ESBL genes [40]. We hypothesize backyard farms in more natural (or at least less anthropogenic) settings are more prone to wildlife exposure, as birds are more exposed to the elements and water treatment could differ in various farm locations [52, 53]. Collectively, we need a better understanding of the factors that lead ESBL *E*. *coli* to be so much more common on backyard farms if we are going to reduce the prevalence of these microbes or even mitigate their exposures. Also unclear is whether the high prevalence of ESBL *E*. *coli* on backyard farms is relatively new. The first accounts of ESBL pathogens in the United States were in the late 1980s and do not preclude even earlier records [54]. Overall, factors not related to direct antimicrobial use on broilers may facilitate the spread of ESBL *E*. *coli*.

There are multiple different types of ESBLs. In our study, whole genome sequencing revealed that all ESBL *E*. *coli* isolates contained *bla*_CTX-M_ type genes. CTX-M stands for CefoTaXime-hydrolyzing beta-lactamase isolated in Munich (M); this type is known to preferentially hydrolyze cefotaxime relative to ceftazidime, both third generation cephalosporins [1, 55]. Our genomic result is consistent with our phenotypic results, as all of our isolates displayed cefotaxime resistance but around 16% displayed resistance to ceftazidime (n = 17/107). *bla*_CTX-M_ variants represent unique molecular lineages. As revealed by whole-genome sequencing, the most common *bla*_CTX-M_ type found in this study was *bla*_CTX-M-1_ (n = 61/107). Other studies of broilers have also found *bla*_CTX-M_ type to be the most common in broiler chickens, especially bla_CTX-M-1_ [7, 56, 57]. Variants *bla*_CTX-M-55_ and *bla*_CTX-M-65_ were also detected and have been reported to be found in retail chicken meat in South Korea [58]. One U.S. study found *bla*_CTX-M-65_ prevalent in *Salmonella* in chicken retail meat [59]. *bla*_CTX-M-15_ was noted in just a few isolates in our study; however, this variant appears to be dominating worldwide [55, 60]. It has been associated with chickens in other countries, but is of concern in both human and companion animal hospital settings [51, 56, 61, 62]. It is possible that backyard chickens are one context in which this ESBL type may move out of anthropogenic settings and into other species and habitats.

Identification of *bla*_CTX-M-15_, potentially gives greater insight into the epidemiology of this pathogen in terms of a potential source and emphasizes the ease of contamination. *bla*_CTX-M-15_ type *Enterobacteriaceae* has been noted as a concern in hospitals in the Southeastern U.S., where our research was conducted [62]. Chen and colleagues in 2014 conducted a study in which most of the patients presented with ESBL presented with ESBL infection upon hospital arrival, indicating they did not acquire it from their current visit [62]. This would indicate acquisition of ESBL *Enterobacteriaceae* from outside the hospital; however, most of these patients in had also been hospitalized within the past year prior to the study [62], leaving open the possibility that they acquired the pathogen on a previous visit. It is still ambiguous as to where ESBL *E*. *coli* originates, but it appears to fester in a variety of environments. Further research is necessary to delineate if hospitals could potentially be a source contributing to the transmission of ESBL pathogens to humans (and eventually backyard broilers). Given the prevalence of ESBL pathogens in our study, hospitals seem unlikely to be the sole source of these pathogens. However, farm owners enter the bird’s immediate environment more regularly than is done for commercial birds and could, in some cases, be vectoring pathogens to their birds. We did not ask farm owners if any members of the family had been in a hospital or other intensive healthcare setting recently, but this could be an important question for future studies. Overall, the *bla*_CTX-M_ family is globally emerging as the most prominent ESBL type and is important to consider especially given the threat of transmission between non-human animals and humans, or vice versa [6, 63–65]. Our study establishes the presence of *bla*_CTX-M_ type ESBLs in backyard broiler farms in a portion of the Southeastern U.S. and raises concern for zoonotic transmission in a home environment.

Our study revealed expected resistance to penicillin and 3^rd^ generation cephalosporins, as resistance is commonly associated with ESBL pathogens [1]. However, we also found resistance to 4^th^ generation cephalosporin, cefepime, which is not typically included in the ESBL resistance repertoire. Our study revealed many susceptible dose-dependent and few resistant isolates to cefepime. Concern of resistance to cefepime has been documented and studies show cefepime should not be considered a treatment option of ESBL infections considering this growing resistance [63, 66]. Though we attribute a lot of the resistance phenotypically shown by our isolates to be from *bla*_CTX-M_ enzyme production, we also had overlapping *ampC* and *TEM-1* genes detected. These genes overlapped with *bla*_CTX-M_ type genes, which has been seen in other studies as well, potentially contributing to the phenotypic resistance profile [7, 64]. Though resistance is expected with these genes, it should not be taken lightly especially when resistance occurs in the later generations that cover a broader range of infection, such as extended-spectrum cephalosporins. Later generation cephalosporins are valuable and resistance to them is a public health threat. Our study confirms the presence of not just ESBLs, but cefepime resistant isolates in backyard broiler farm systems.

We also tested for two clinically important antimicrobial classes, ciprofloxacin (fluoroquinolone) and gentamicin (aminoglycoside). These antimicrobials are not in the beta-lactam group. They are widely used in treating urinary tract infections (UTI’s) and ESBL infections [67]. In our study, phenotypic resistance to ciprofloxacin was noted in three isolates as was gentamicin, though not in the same isolates. Concern of resistance to these antimicrobials has been noted especially when treating ESBL pathogens [67–69]. The ability to use non-beta-lactam antibiotics in cases of ESBL infection offers clinicians a solution just short of using last resort drugs; however, it appears growing resistance may shut down this option with increasing frequency [70]. Potential co-selection of aminoglycoside and fluoroquinolone resistance with ESBL pathogens has been noted and is a concerning public health burden as it limits treatment options [71, 72]. Though resistance to these particular antimicrobials was not widespread in this study, prevalence of antimicrobial resistance genes particularly to aminoglycoside were noted on plasmids, which are known for spread of AMR genes, in particular ESBL genes [73].

Plasmids could play a role in mediating the prevalence of antimicrobial resistance genes in environments, even in the absence of antimicrobial use. Our research found that all *bla*_CTX-M-1_ genes were carried on plasmids and the majority of *bla*_CTX-M-65_ were carried on plasmids. It is well documented that the exchange of plasmids has facilitated the emergence of ESBL, particularly *bla*_CTX-M_ type ESBL [6, 15, 74]. The most common plasmid found in this study was the IncFIB(AP001918)_1 plasmid. IncF plasmids are of particular concern in *bla*_CTX-M_ gene spread in *Enterobacteriaceae* [56, 75]. Previous studies have revealed that plasmids associated with ESBL pathogens are similar between human and broiler sources; this is important to consider when it comes to zoonotic spread between the two species [15, 76]. This further emphasizes a need for an interdisciplinary approach to this issue as plasmids enhance the spread of ESBL genes between a variety of sources, as they appear to not be limited to just broilers or humans [76]. Overall, a better understanding of the plasmids involved is important for understanding the zoonotic spread of these critical ESBL genes between broilers and humans.

Carbapenems are known as a last resort antimicrobial that is effective in multidrug resistant infections [77]. With the rise of ESBL pathogens, the increased need for carbapenem use is predicted to lead to eventual resistance to this “last resort” drug [1]. We did not observe resistance to carbapenems in our ESBL *E*. *coli* isolates (imipenem and meropenem). We also observed that all isolates were susceptible to piperacillin tazobactam, a penicillin with beta-lactamase inhibitory that can be used in case of ESBL infection [78]. Surveillance needs to continue in order to monitor potential prevalence in disease causing microbes and understand the spread of such important resistance.

## Conclusion

Our study establishes the prevalence of *bla*_CTX-M_ type ESBL *E*. *coli* in backyard broiler production systems in a portion of the Southeastern United States. While *bla*_CTX-M_ type ESBL *E*. *coli* were also found in commercial farms, the prevalence was significantly lower compared to backyard farms. While we believe further research is necessary to determine why, we feel a couple major contributing factors could be zoonotic transfer from other non-human animal species or humans on the farm as well as bird exposures prior to farm arrival. This study also supports concerns of increasing resistance to fourth generation cephalosporin, cefepime, as we found susceptible dose-dependent and resistant isolates. Through our work, we highlight the importance of considering zoonotic transmission between broilers and humans, especially with the growing popularity of backyard poultry ownership in the United States. Overall, we emphasize the need to continue surveillance and consider factors that could be exacerbating the prevalence of these pathogens in the broiler farm environment.

## References

[1] Castanheira M, Simner PJ, Bradford PA. Extended-spectrum β-lactamases: an update on their characteristics, epidemiology and detection. JAC-antimicrobial resistance. 2021;3(3):dlab092–dlab. doi: 10.1093/jacamr/dlab092 34286272 PMC8284625

[2] Eriksen NL. Extended-spectrum (second- and third-generation) cephalosporins. Obstetrics and Gynecology Clinics of North America. 1992;19(3):461–74. . 1436924

[3] CDC. Antibiotic Resistance Threats in the United States. 2019. p. 1–150.

[4] Shah DH, Board MM, Crespo R, Guard J, Paul NC, Faux C. The occurrence of Salmonella, extended‐spectrum β‐lactamase producing Escherichia coli and carbapenem resistant non‐fermenting Gram‐negative bacteria in a backyard poultry flock environment. Zoonoses and public health. 2020;67(6):742–53. doi: 10.1111/zph.12756 32710700

[5] Tansawai U, Walsh TR, Niumsup PR. Extended spectrum ß-lactamase-producing Escherichia coli among backyard poultry farms, farmers, and environments in Thailand. Poultry science. 2019;98(6):2622–31. doi: 10.3382/ps/pez009 30690545

[6] Dierikx CM, van der Goot JA, Smith HE, Kant A, Mevius DJ, info:eu rdn, et al. Presence of ESBL/AmpC -Producing Escherichia coli in the Broiler Production Pyramid: A Descriptive Study. PloS one. 2013;8(11):e79005–e. doi: 10.1371/journal.pone.0079005 24244401 PMC3820706

[7] Saliu EM, Vahjen W, Zentek J. Types and prevalence of extended-spectrum beta-lactamase producing Enterobacteriaceae in poultry. Animal Health Research Reviews. 2017;18(1):46–57. doi: 10.1017/S1466252317000020 28641596

[8] Brower CH, Mandal S, Hayer S, Sran M, Zehra A, Patel SJ, et al. The Prevalence of Extended-Spectrum Beta-Lactamase-Producing Multidrug-Resistant *Escherichia coli* in Poultry Chickens and Variation According to Farming Practices in Punjab, India. Environmental Health Perspectives. 2017;125(7):1–10. 10.1289/EHP292. 28749780 PMC5744676

[9] USDA. Chicken’s popularity makes it the most consumed U.S. meat 2016.

[10] Shahbandeh M. Meat consumption worldwide from 1990 to 2021, by meat type 2023. Available from: https://www.statista.com/statistics/274522/global-per-capita-consumption-of-meat/#:~:text=Meat%20consumption%20worldwide%201990%2D2021%2C%20by%20type&text=In%202021%2C%20around%20132.3%20million,consumed%20type%20of%20meat%20globally.

[11] Elkhoraibi C, Blatchford RA, Pitesky ME, Mench JA. Backyard chickens in the United States: A survey of flock owners. Poultry science. 2014;93(11):2920–31. doi: 10.3382/ps.2014-04154 25193256

[12] USDA. Urban Chicken Ownership in Four U.S. Cities Fort Collins, CO 2013. Available from: https://www.aphis.usda.gov/animal_health/nahms/poultry/downloads/poultry10/Poultry10_dr_Urban_Chicken_Four_1.pdf.

[13] FAO. Markets and Trade. Available from: https://www.fao.org/poultry-production-products/socio-economic-aspects/markets-and-trade/en/#:~:text=Poultry%20tends%20to%20be%20cheaper,%2Dscale%2C%20specialized%20commercial%20producers.

[14] USDA. Part I: Reference of Health and Management of Backyard/Small Production Flocks in the United States, 2004 Fort Collins, CO 2005. Available from: https://www.aphis.usda.gov/animal_health/nahms/poultry/downloads/poultry04/Poultry04_dr_PartI.pdf.

[15] van Hoek A, Dierikx C, Bosch T, Schouls L, van Duijkeren E, Visser M. Transmission of ESBL-producing Escherichia coli between broilers and humans on broiler farms. Journal of Antimicrobial Chemotherapy. 2020;75(3):543–9. doi: 10.1093/jac/dkz507 31800052

[16] Shah DH, Board MM, Crespo R, Guard J, Paul NC, Faux C. The occurrence of Salmonella, extended-spectrum B-lactamase producing Escherichia coli and carbapenem resistant non-fermenting Gram-negative bacteria in a backyard poultry flock environment. Zoonoses and Public Health. 2020;67(6):742–53. doi: 10.1111/zph.12756 32710700

[17] NARMS. National Antimicrobial Resistance Monitoring System (NARMS) Retail Meat Surveillance Laboratory Protocol. 2021.

[18] Jacob ME, Keelara S, Aidara-Kane A, Matheu Alvarez JR, Fedorka-Cray PJ. Optimizing a Screening Protocol for Potential Extended-Spectrum Beta-Lactamase *Escherichia coli* on MacConkey Agar for Use in a Global Surveillance Program. Journal of Clinical Microbiology. 2020;58(9). doi: 10.1128/JCM.01039-19 32434784 PMC7448649

[19] NARMS. The National Antimicrobial Resistance Monitoring System Manual of Laboratory Methods. Fourth ed 2020.

[20] CLSI. CLSI M100 ED33:2023. 2023.

[21] NARMS. Antibiotics Tested by NARMS. 2019.

[22] Hull DM, Harrell E, van Vliet AHM, Correa M, Thakur S. Antimicrobial resistance and interspecies gene transfer in Campylobacter coli and Campylobacter jejuni isolated from food animals, poultry processing, and retail meat in North Carolina, 2018–2019. PloS one. 2021;16(2):e0246571–e. doi: 10.1371/journal.pone.0246571 33571292 PMC7877606

[23] Bankevich A, Nurk S, Antipov D, Gurevich A, Dvorkin M, Kulikov A, et al. SPAdes: A New Genome Assembly Algorithm and Its Applications to Single-Cell Sequencing. Journal of Computational Biology. 2012;19(5). doi: 10.1089/cmb.2012.0021 22506599 PMC3342519

[24] Gurevich A, Saveliev V, Vyahhi N, Tesler G. QUAST: quality assessment tool for genome assemblies. Bioinformatics. 2013;29(8):1072–5. doi: 10.1093/bioinformatics/btt086 23422339 PMC3624806

[25] Seemann T. ABRicate 2020. Available from: https://github.com/tseemann/abricate.

[26] Alcock BP, Raphenya AR, Lau TTY, Tsang KK, Bouchard M, Edalatmand A, et al. CARD 2020: antibiotic resistome surveillance with the comprehensive antibiotic resistance database. Nucleic Acids Res. 2020;48(D1):D517–D25. doi: 10.1093/nar/gkz935 ; PubMed Central PMCID: PMC7145624. 31665441 PMC7145624

[27] Carattoli A, Zankari E, Garcia-Fernandez A, Larsen M, Lund O, Villa L, et al. *In Silico* Detection and Typing of Plasmids using PlasmidFinder and Plasmid Multilocus Sequence Typing. Antimicrobial Agents and Chemotherapy. 2014;58(7):3895–903. 10.1128/aac.02412-14. 24777092 PMC4068535

[28] Chen L, Zheng D, Liu B, Yang J, Jin Q. VFDB 2016: hierarchical and refined dataset for big data analysis—10 years on. Nucleic Acids Res. 2016;44(D1):D694–7. doi: 10.1093/nar/gkv1239 26578559 PMC4702877

[29] Doster E, Lakin S, Dean C, Wolfe C, Young J, Boucher C, et al. MEGARes 2.0: a database for classification of antimicrobial drug, biocide and metal resistance determinants in metagenomic sequence data. Nucleic Acids Res. 2020;48:561–9. 31722416 10.1093/nar/gkz1010 PMC7145535

[30] Feldgarden M, Brover V, Haft D, Prasad A, Slotta D, Tolstoy I, et al. Validating the AMRFinder Tool and Resistance Gene Database by Using Antimicrobial Resistance Genotype-Phenotype Correlations in a Collection of Isolates. Antimicrobial Agents and Chemotherapy. 2019;63(11):1–19. 10.1128/aac.00483-19. PMC6811410 31427293

[31] Bertels F, Silander O, Pachkov M, Rainey P, van Nimwegen E. Automated Reconstruction of Whole-Genome Phylogenies from Short-Sequence Reads. Molecular Biology and Evolution. 2014;31(5):1077–88. doi: 10.1093/molbev/msu088 24600054 PMC3995342

[32] Letunic I, Bork P. Interactive Tree Of Life (iTOL) v5: an online tool for phylogenetic tree display and annotation. Nucleic Acids Research. 2021;49(W1):W293–W6. doi: 10.1093/nar/gkab301 33885785 PMC8265157

[33] CDC. Epi Info ^TM^ StatCalc. 2022.

[34] Bui T, Preuss CV. StatPearls. Treasure Island (FL): StatPearls Publishing; 2022.

[35] Rodloff A, Bauer T, Ewig S, Kujath P, Muller E. Susceptible, Intermediate, and Resistant- The Intensity of Antibiotic Action. Deutsches Arzteblatt International. 2008;105(39):657–62. doi: 10.3238/arztebl.2008.0657 19626213 PMC2701059

[36] Jacoby GA. AmpC beta-Lactamases. Clinical Microbiology Reviews. 2009;22(1):161–82. doi: 10.1128/CMR.00036-08 19136439 PMC2620637

[37] Gonzalez I, Georgiou M, Alcaide F, Balas D, Linares J, de la Campa AG. Fluoroquinolone Resistance Mutations in the *parC*, *parE*, and *gyrA* Genes of Clinical Isolates of Viridans Group Streptococci. Antimicrobial Agents and Chemotherapy. 1998;42(11):2792–8. doi: 10.1128/aac.42.11.2792 9797205 PMC105945

[38] Juraschek K, Borowiak M, Tausch SH, Malorny B, Kasbohrer A, Otani S, et al. Outcome of Different Sequencing and Assembly Approaches on the Detection of Plasmids and Localization of Antimicrobial Resistance Genes in Commensal Escherichia coli. Microorganisms. 2021;9(3):1–19. doi: 10.3390/microorganisms9030598 33799479 PMC8000739

[39] Bush K, Bradford PA. Beta-Lactams and Beta-Lactamase Inhibitors: An Overview. Cold Spring Harbor Perspectives in Medicine. 2016;6(8):1–22. doi: 10.1101/cshperspect.a025247 27329032 PMC4968164

[40] Borges CA, Tarlton NJ, Riley LW. Escherichia coli from Commercial Broiler and Backyard Chickens Share Sequence Types, Antimicrobial Resistance Profiles, and Resistance Genes with Human Extraintestinal Pathogenic Escherichia coli Foodborne Pathogens and Disease. 2019;16(12):1–10. 10.1089/fpd.2019.2680. 31411497

[41] van den Bogaard AE, Longdon N, Driessen C, Stobberingh E. Antibiotic resistance of faecal *Escherichia coli* in poultry, poultry farmers and poultry slaughterers. Journal of Antimicrobial Chemotherapy. 2001;47(6):763–71. https://doi-org.prox.lib.ncsu.edu/10.1093/jac/47.6.763. 11389108 10.1093/jac/47.6.763

[42] Leverstein-van Hall MA, Dierikx CM, Stuart CJ, Voets GM, van den Munckhof MP, van Essen-Zandbergen A, et al. Dutch patients, retail chicken meat and poultry share the same ESBL genes, plasmids and strains. Clinical Microbiology and Infeciton. 2011;17(6):873–80. 10.1111/j.1469-0691.2011.03497.x. 21463397

[43] Subramanya SH, Bairy I, Nayak N, Amberpet R, Padukone S, Metok Y, et al. Detection and characterization of ESBL-producing Enterobacteriaceae from the gut of healthy chickens, Gallus gallus domesticus in rural Nepal: Dominance of CTX-M-15-non-ST131 Escherichia coli clones. PLoS One. 2020;15(5):1–15. doi: 10.1371/journal.pone.0227725 32469888 PMC7259619

[44] Samanta I, Joardar SN, Mahanti A, Bandyopadhyay S, Sar TK, Dutta TK. Approaches to characterize extended spectrum beta-lactamase/beta-lactamase producing *Escherichia coli* in healthy organized *vis-a-vis* backyard farmed pigs in India. Infection, Genetics and Evolution. 2015;36:224–30. 10.1016/j.meegid.2015.09.021. 26423671

[45] Al-Marri T, Al-Marri A, Al-Zanbaqi R, Al Ajmi A, Fayez M. Multidrug resistance, biofilm formation, and virulence genes of Escherichia coli from backyard poultry farms. Veterinary World. 2021;14(11):2869–77. doi: 10.14202/vetworld.2021.2869-2877 35017833 PMC8743762

[46] Osman KM, Kappell AD, Elhadidy M, ElMougy F, Abd El-Ghany WA, Orabi A, et al. Poultry hatcheries as potential reservoirs for antimicrobial-resistant Escherichia coli: A risk to public health and food safety. Scientific Reports. 2018;8(5859):1–14. doi: 10.1038/s41598-018-23962-7 29643424 PMC5895583

[47] Doyle MP, Erickson MC. Reducing the Carriage of Foodborne Pathogens in Livestock and Poultry. Poultry Science. 2006;85(6):960–73. doi: 10.1093/ps/85.6.960 16776463

[48] Salgado-Caxito M, Benavides JA, Adell AD, Paes AC, Moreno-Switt AI. Global prevalence and molecular characterization of extended-spectrum beta-lactamase producing Escherichia coli in dogs and cats—A scoping review and meta-analysis. One Health. 2021;12:1–15. doi: 10.1016/j.onehlt.2021.100236 33889706 PMC8050393

[49] Dahms C, Hubner N-O, Kossow A, Mellmann A, Dittmann K, Kramer A. Occurrence of ESBL-Producing Escherichia coli in Livestock and Farm Workers in Mecklenburg-Western Pomerania, Germany. PLoS One. 2015;10(11):1–13. doi: 10.1371/journal.pone.0143326 26606146 PMC4659621

[50] Ibekwe A, Durso L, Ducey TF, Oladeinde A, Jackson CR, Frye JG, et al. Diversity of Plasmids and Genes Encoding Resistance to Extended-Spectrum Beta-Lactamase in Escherichia coli from Different Animal Sources. Microorganisms. 2021;9(5):1–16. 10.3390/microorganisms9051057. PMC8153348 34068339

[51] Timofte D, Maciuca IE, Williams NJ, Wattret A, Schmidt V. Veterinary Hospital Dissemination of CTX-M-15 Extended-Spectrum Beta-Lactamase-Producing *Escherichia coli* ST410 in the United Kingdom. Microbial Drug Resistance. 2016;22(7). 10.1089/mdr.2016.0036. PMC5073239 27314838

[52] CDC. Water Treatment 2022. Available from: https://www.cdc.gov/healthywater/drinking/public/water_treatment.html#:~:text=Water%20treatment%20differs%20by%20community,that%20enters%20the%20treatment%20plant.

[53] Strosnider H, Kennedy C, Monti M, Yip F. Rural and Urban Differences in Air Quality, 2008–2012, and Community Drinking Water Quality, 2010–2015—United States. Morbidity and Mortality Weekly Report. 2017;66(13):1–10. doi: 10.15585/mmwr.ss6613a1 28640797 PMC5829865

[54] Rice LB, Willey SH, Papanicolaou GA, Medeiros AA, Eliopoulos GM, Moellering RCJ, et al. Outbreak of Ceftazidime resistance caused by extended-spectrum beta-lactamases at a Massachusetts chronic-care facility. Antimicrobial Agents and Chemotherapy. 1990;34(11):2193–9. doi: 10.1128/AAC.34.11.2193 2073110 PMC172022

[55] Canton R, Gonzalez-Alba JM, Glan JC. CTX-M Enzymes: Origin and Diffusion. Frontiers in Microbiology. 2012;3(110):1–19. doi: 10.3389/fmicb.2012.00110 22485109 PMC3316993

[56] Seo KW, Lee YJ. The occurrence of CTX-M-producing *E*. *coli* in the broiler parent stock in Korea. Poultry Science. 2021;100(2):1008–15. doi: 10.1016/j.psj.2020.09.005 33518059 PMC7858018

[57] Al-Mustapha AI, Raufu IA, Ogundijo OA, Odetokun IA, Tiwari A, Brouwer MSM, et al. Antibiotic resistance genes, mobile elements, virulence genes, and phages in cultivated ESBL-producing *Escherichia coli* of poultry origin in Kwara State, North Central Nigeria. International Journal of Food Microbiology. 2023;389(16):1–9. 10.1016/j.ijfoodmicro.2023.110086. 36738714

[58] Park H, Kim J, Ryu S, Jeon B. Predominance of bla CTX-M-65 and blaCTX-M-55 in extended-spectrum beta-lactamase-producing Escherichia coli from raw retail chicken in South Korea. 2019;17:216–20. 10.1016/j.jgar.2019.01.005. 30658198

[59] Brown AC, Chen JC, Francois Watkins LK, Campbell D, Folster JP, Tate H, et al. CTX-M-65 Extended-Spectrum Beta-Lactamase-Producing *Salmonella enterica* Serotype Infantis, United States. Emerging Infectious Diseases. 2018;24(12):2284–91. doi: 10.3201/eid2412.180500 30457533 PMC6256390

[60] Bevan ER, Jones AM, Hawkey PM. Global epidemiology of CTX-M beta-lactamases: temporal and geographical shifts in genotype. Journal of Antimicorbial Chemotherapy. 2017;72(8):2145–55. 10.1093/jac/dkx146. 28541467

[61] Maciuca IE, Williams NJ, Tuchilus C, Dorneanu O, Guguianu E, Carp-Carare C, et al. High Prevalence of Escherichia coli-Producing CTX-M-15 Extended-Spectrum Beta-Lactamases in Poultry and Human Clinical Isolates in Romania. Microbial Drug Resistance. 2015;21(6):651–62. doi: 10.1089/mdr.2014.0248 25734920

[62] Chen LF, Freeman JT, Nicholson B, Keiger A, Lancaster S, Joyce M, et al. Widespread Dissemination of CTX-M-15 Genotype Extended-Spectrum-Beta-Lactamase-Producing *Enterobacteriaceae* among Patients Presenting to Community Hospitals in the Southeastern United States. Antimicrobial Agents and Chemotherapy. 2014;58(2):1200–2. doi: 10.1128/AAC.01099-13 24247126 PMC3910860

[63] Isler B, Harris P, Stewart A, Paterson D. An update on cefepime and its future role in combination with novel beta-lactamase inhibitors for MDR Enterobacterales and Pseudomonas aeruginosa Journal of Antimicrobial Chemotherapy. 2020;76(3):550–60. 10.1093/jac/dkaa511. 33332545

[64] Misumi W, Magome A, Okuhama E, Uchimura E, Tamamura-Andoh Y, Watanabe Y, et al. CTX-M-55-type ESBL-producing fluoroquinolone-resistant Escherichia coli sequence type 23 repeatedly caused avian colibacillosis in Kagoshima Prefecture, Japan. Journal of Global Antimicrobial Resistance. 2023;35:325–31. doi: 10.1016/j.jgar.2023.10.015 37918785

[65] Rossolini GM D’Andrea MM, Mugnaioli C. The spread of CTX-M-type extended-spectrum beta-lactamases. Clinical Microbiology and Infection. 2008;14(1):31–41. doi: 10.1111/j.1469-0691.2007.01867.x 18154526

[66] Patel HB, Lusk KA, Cota JM. The Role of Cefepime in the Treatment of Extended-Spectrum Beta-Lactamase Infections. Journal of Pharmacy Practice. 2017;32(4):458–63. doi: 10.1177/0897190017743134 29166830

[67] Mwakyoma AA, Kidenya BR, Minja CA, Mushi MF, Sandeman A, Sabiti W, et al. Allele distribution and phenotypic resistance to ciprofloxacin and gentamicin among extended-spectrum beta-lactamase-producing Escherichia coli isolated from the urine, stool, animals, and environments of patients with presumptive urinary tract infection in Tanzania. Frontiers Antibiotics. 2023;2(2023):1–11. doi: 10.3389/frabi.2023.1164016 PMC11732152 39816664

[68] Tolun V, Kucukbasmaci O, Torumkuney-Akbulut D, Catal C, Ang-Kucuker M, Aug O. Relationship between ciprofloxacin resistance and extended-spectrum beta-lactamase production in *Escherichia coli* and *Klebsiella pneumoniae* strains. Clinical Microbiology and Infection. 2004;10(1):72–5. 10.1111/j.1469-0691.2004.00723.x. 14706090

[69] Seok H, Cha MK, Kang C-I, Cho SY, Ki SH, Ha EY, et al. Failure of Ciprofloxacin Therapy in the Treatment of Community-Acquired Acute Pyelonephritis caused by In-Vitro Susceptible Escherichia coli Strain Producing CTX-Type Extended-Spectrum beta-Lactamase. Infection and Chemotherapy. 2018;50(4):357–61. doi: 10.3947/ic.2018.50.4.357 30600660 PMC6312905

[70] Wiener ES, Heil EL, Hynicka LM, Johnson KJ. Are Fluoroquinolones Appropriate for the Treatment of Extended-Spectrum beta-Lactamase-Producing Gram-Negative Bacilli? Journal of Pharmacy Technology. 2016;32(1):16–21. doi: 10.1177/8755122515599407 34860959 PMC5998409

[71] Canton R, Ruiz-Garbajosa P. Co-resistance: an opportunity for the bacteria and resistance genes. Current Opinion in Pharmacology. 2011;11(5):477–85. doi: 10.1016/j.coph.2011.07.007 21840259

[72] Hussain HI, Aqib AI, Seleem MN, Shabbir MA, Hao H, Iqbal Z, et al. Genetic Basis of Molecular Mechanisms in beta-lactam Resistant Gram-negative Bacteria. Microbial Pathogenesis. 2021;158:1–26. doi: 10.1016/j.micpath.2021.105040 34119627 PMC8445154

[73] Gekenidis M-T, Klaui A, Smalla K, Drissner D. Trasferable Extended-Spectrum Beta-Lactamase (ESBL) Plasmids in Enterobacteriaceae from Irrigation Water. Microorganisms. 2020;8(7):1–14. doi: 10.3390/microorganisms8070978 32629840 PMC7409067

[74] Chowdhury M, Bardhan R, Pal S, Banerjee A, Batabyal K, Joardar SN, et al. Comparative occurrence of ESBL/AmpC beta-lactamase- producing Escherichia coli and Salmonella in contract farm and backyard broilers. 2022;74(1):53–62. doi: 10.1111/lam.13581 34618368

[75] Bortolaia V, Guardabassi L, Trevisani M, Bisgaard M, Venturi L, Bojesen AM. High Diversity of Extended-Spectrum Beta-Lactamases in *Escherichia coli* Isolates from Italian Broiler Flocks. Antimicrobial Agents and Chemotherapy. 2010;54(4):1623–6. 20100875 10.1128/AAC.01361-09 PMC2849389

[76] Kurittu P, Khakipoor B, Aarnio M, Nykasenoja S, Brouwer M, Myllyniemi A-L, et al. Plasmid-Borne and Chromosomal ESBL/AmpC Genes in Escherichia coli and Klebsiella pneumoniae in Global Food Products Frontiers in Microbiology. 2021;12:1–19. doi: 10.3389/fmicb.2021.592291 33613476 PMC7886708

[77] Potter RF D’Souza AW, Dantas G. The rapid spread of carbapenem-resistant Enterobacteriaeae. Drug Resistant Updates. 2016;29. doi: 10.1016/j.drup.2016.09.002 27912842 PMC5140036

[78] Hoashi K, Hayama B, Suzuki M, Sakurai A, Takehana K, Enokida T, et al. Comparison of the Treatment Outcome of Piperacillin-Tazobactam versus Carbapenems for Patients with Bacteremia Caused by Extended-SPectrum Beta-Lactamase-Producing *Escherichia coli* in Areas with Low Frequency Coproduction of OXA-1: a Preliminary Analysis. Clinical Microbiology. 2022;10(4):1–10. 10.1128/spectrum.02206-22. PMC9430612 35916524

